# Constitutive expression of a novel antimicrobial protein, *Hcm1*, confers resistance to both Verticillium and Fusarium wilts in cotton

**DOI:** 10.1038/srep20773

**Published:** 2016-02-09

**Authors:** Zhiyuan Zhang, Jun Zhao, Lingyun Ding, Lifang Zou, Yurong Li, Gongyou Chen, Tianzhen Zhang

**Affiliations:** 1 National Key Laboratory of Crop Genetics & Germplasm Enhancement, Cotton Research Institute, Nanjing Agricultural University, Nanjing 210095, P. R. China; 2 School of Agriculture and Biology, Shanghai Jiao Tong University/Key Laboratory of Urban (South) by Ministry of Agriculture, Shanghai, China

## Abstract

Fusarium and Verticillium wilts, two of the most important diseases in cotton, pose serious threats to cotton production. Here we introduced a novel antimicrobial protein *Hcm1*, which comprised harpin protein from *Xanthomonas oryzae* pv. *oryzicola* (*Xoc*), and the chimeric protein, cecropin A-melittin, into cotton. The transgenic cotton lines with stable *Hcm1* expression showed a higher resistance to Verticillium and Fusarium wilts both in greenhouse and field trials compared to controls. *Hcm1* enabled the transgenic cotton to produced a microscopic hypersensitive response (micro-HR), reactive oxygen species (ROS) burst, and caused the activation of pathogenesis-related (PR) genes in response to biotic stress, indicating that the transgenic cotton was in a primed state and ready to protect the host from pathogenic infection. Simultaneously, Hcm1 protein inhibited the growth of *Verticillium dahliae* (*V. dahliae*) and *Fusarium oxysporum* (*F. oxysporum*) *in vitro*. The spread of fungal biomass was also inhibited *in vivo* since the *V. dahliae* biomass was decreased dramatically in transgenic cotton plants after inoculation with *V. dahliae*. Together, these results demonstrate that *Hcm1* could activate innate immunity and inhibit the growth of *V. dahliae* and *F. oxysporum* to protect cotton against Verticillium and Fusarium wilts.

Fungal disease is a major threat to both crop yields and global food security^1^,^2^,^3^. Fusarium wilt and Verticillium wilt, also known as vascular wilt, pose the largest threat of disease to most economically important crops, such as tomato and cotton. In particular, Verticillium wilt has been reported in most cotton-growing areas, and is the most important disease of cotton in the world^4^. About half of the cotton cultivating area in China was subjected to this disease in 2009 and 2010 (National Cotton Council of America-Disease Database). Traditional methods of pathogen control rely heavily on two methods: biological control measures such as cultivar choice and crop rotation and chemical control. Intensive plant breeding and chemical controls allow farmers to overcome many common plant diseases. However, effective fungicides or alternative methods for controlling *Verticillium dahliae* (*V. dahliae*) infection is still lacking^5^. Transgenic technology for the control of insect herbivores and weeds offers an alternative approach to enhance plant resistance to fungal pathogens^6^. Genetic engineering techniques are the most economic and effective means for managing Verticillium wilt^7^. Some genes have been reported to confer resistance to Verticillium wilt in cotton. The Verticillium resistance genes, *Ve1*, were cloned using a map-based cloning strategy in tomato plants^8^. Overexpression of *Gbve1*, a cotton gene homologous to the tomato *Ve* gene, endowed transgenic *Arabidopsis* and upland cotton with resistance to both highly aggressive defoliating and non-defoliating isolates of *V. dahliae*^9^. *Baculovirus* anti-apoptotic genes p35 and op-iap could enhance tolerance to Verticillium wilt in transgenic cotton^10^. Zhao *et al.*^11^ reported that overexpression of *GbRLK*, a putative receptor-like kinase gene, improved cotton resistance to Verticillium wilt. Some researchers report that introducing foreign genes to cotton could enhance resistance to both Verticillium and Fusarium wilts. Expression of *Arabidopsis NPR1* in cotton confers significant resistance to multiple pathogens, including *V. dahliae* and *Fusarium oxysporum* (*F. oxysporum*)^12^,^13^. The plant defensin NaD1, which inhibits the growth of fungal pathogens *in vitro*, confers resistance to Fusarium wilt and Verticillium wilt in cotton^6^. *Hpa1*_*Xoo*_, which induces the hypersensitive response (HR), improved cotton resistance to Verticillium wilt and Fusarium wilt^14^.

The HR is one component of plant immunity. It is a rapid, local defense-related programmed cell death triggered by the effectors produced by microbial pathogens^15^,^16^. Harpin is a type of pathogen effector that is secreted from bacteria *via* a type-III secretion system (T3SS)^17^. Harpin was first identified as an HR-elicitor^18^. The application of Harpin induces the HR, a reactive oxygen species (ROS) burst^14^,^19^, and activates the expression of HR markers such as *HIN1*^20^ and *HSR203J*^21^, and pathogenesis-related (PR) genes such as *PR1a* and *PR1b*^21^,^22^,^23^ in plants. Plants treated with harpins at an early growth stage show systemic acquired resistance (SAR) to pathogens and insects, and exhibit benefits in both growth and yield^15^,^24^. However, harpins currently used as plant defense-activators have no antimicrobial properties^25^.

Some antimicrobial proteins against pathogens have been identified in insects. Cecropin A, isolated from the hemolymph of the cecropia moth, shows broad spectrum capability of suppressing bacteria, fungi, enveloped viruses, and tumor cells^26^,^27^,^28^. Another protein isolated from bee venom^29^, melittin, has been shown to be active against bacterial and human red blood cells^30^,^31^,^32^. Due to the bacterial suppression activity of melittin, researchers employed genetic engineering techniques to introduce it into plant genomes in order to improve their pathogen resistance. To suppress the hemolytic activity of melittin, an artificial protein was created by joining the α-helix structures of two peptides, cecropin A and melittin. This chimeric protein showed a better antimicrobial spectrum than cecropin A alone, and less hemolytic activity than melittin alone^27^,^30^,^33^. The hybrid peptide can effectively inhibit the proliferation of pathogens in plants^33^,^34^,^35^, however, few hybrid peptides have been shown to activate plant innate immunity.

Compounds designed for use in protecting plants against pathogenic infection are likely to be most effective if they activate innate plant immunity as well as possess antimicrobial activity^36^. In a previous study, a novel chimeric protein, *Hcm1*, consisting of *Hpa1*_*Xoc*_ joined to the active domains of cecropin A-melittin, was constructed. It not only elicited an HR in tobacco, but also effectively inhibited the growth of Gram-negative and Gram-positive bacteria *in vitro*. Plants sprayed with Hcm1 or Hpa1_Xoc_ protein show high resistance to multiple pathogens; exhibiting a broad-spectrum disease resistance. Moreover, the disease resistance of plants sprayed with Hcm1 protein is better than that of plants sprayed with Hpa1_Xoc_ protein, showing that Hpa1_Xoc_ and cecropin A-melittin both contribute to disease resistance^22^. In the present study, *Hcm1* driven by the CaMV35S promoter was transformed into *Gossypium hirsutum* (*G. hirsutum*) acc. W0 using the *Agrobacterium*-mediated method. *Hcm1* was found to confer resistance to a variety of diseases in cotton, including Verticillium wilt and Fusarium wilt, in both greenhouse and field conditions. *Hcm1-*transformed plants demonstrated a micro-HR and an increase in the expression of PR genes in response to biotic stress. The biomass of *V. dahliae* in transgenic plants was also lower than that in the parent W0 plants. Our results showed that constitutive expression of *Hcm1* in cotton plants increased their resistance to two devasting diseases: Verticillium wilt and Fusarium wilt.

## Results

### Generation of *Hcm1*-expressing cotton plants

**Figure 1:**
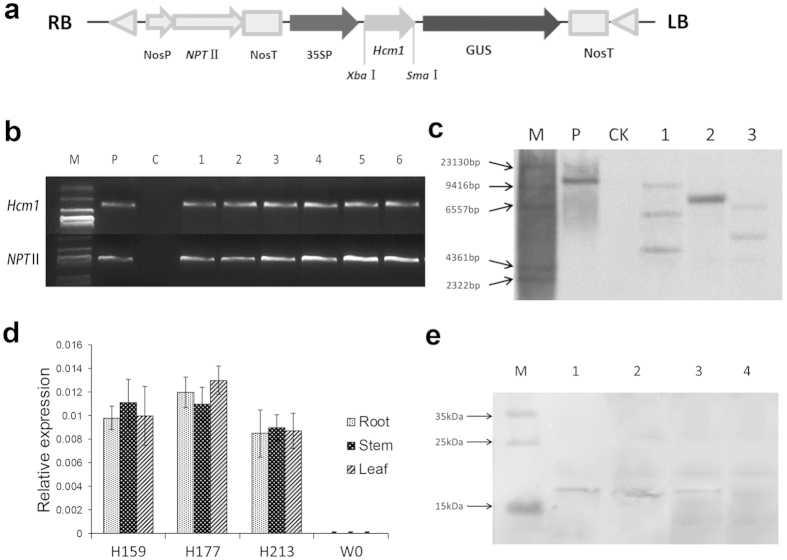
Molecular analysis of *Hcm1* in transgenic plants. (**a**) Schematic representation of recombinant plasmid pBI121-35S::*Hcm1*-*NPT*II. RB and LB represent the right and left borders of T-DNA, respectively. (**b**) PCR analysis of the transgenic plants to detect the 35S: *Hcm1* and the *NPT*II genes. M: Marker DL2000; P: plasmid as positive control; C: non-transformed plant W0; Lanes 1–6: positive transgenic plants. (**c**) Southern blot analysis of *Hcm1* insertions in transgenic lines. Genomic DNA was digested with *Eco*RΙ and hybridized with a 0.75-kb fragment of *NPT*II. M: Marker, P: positive control pBI121, CK: non-transformed W0 plant, lanes 1–3: T6 generation homozygous transgenic lines H159, H177, and H213. (**d**) qRT-PCR analysis of expression levels of *Hcm1* in roots, stems and leaves of *Hcm1*-transformed (lines H159, H177, and H213) and parent W0 plants. Error bars represent the standard deviation of triplicate experiments, and the *EF-1α* gene was amplified as a control. (**e**) Western blot analysis of *Hcm1* in transgenic plants. M: PageRuler^TM^ Prestained Protein Ladder, lanes 1–4: Three T6 generation homozygous transgenic lines (H159, H177, and H213) and parent W0.

In order to improve disease resistance in cotton, a binary transformation vector carrying an *Hcm1* gene cassette (CaMV35S promoter-*Hcm1* ORF-Nos terminator), designated pBI121-*Hcm1* (Fig. 1a), was introduced into *G. hirsutum* acc. W0 using the *Agrobacterium-*mediated transformation method. Primary transgenic plants (T_0_) were allowed to self-pollinate to generate seeds. From generation T_1_ to T_6_, the transgenic lines were screened for their resistance to kanamycin together with the PCR detection of the presence of *NPT*II and *Hcm1* sequence fragments (Fig. 1b). To minimize the effects of the transgenic operation and the insertion location on the chromosome, three homogenous lines, H159, H177, and H213, without observable difference of agronomic characters with parent W0 (Table 1), were selected for further study. Southern blot analysis revealed three, one, and two copies of *Hcm1* in the three homogenous lines, respectively (Fig. 1c). Real-time quantitative reverse transcript PCR (qRT-PCR) analysis found that *Hcm1* was expressed in roots, stems, and leaves of three transgenic lines (Fig. 1d). To further test the expression of the artificial chimeric protein, a multi-clone antibody was generated against Hcm1 protein. Western blotting with the anti-*Hcm1* antibody indicated that the Hcm1 protein at the expected molecular weight of 17 kilodalton (KD) (Fig. 1e) was expressed in the total protein extracted from the leaves of the *Hcm1*-transformed plants. No blotting band was observed in the parent W0 plants. All of these results indicated that *Hcm1* had been successfully transformed into parent W0 plants and constitutively expressed in the transgenic plants.

### Resistance of *Hcm1*-transformed cotton plants to Fusarium wilt

**Figure 2:**
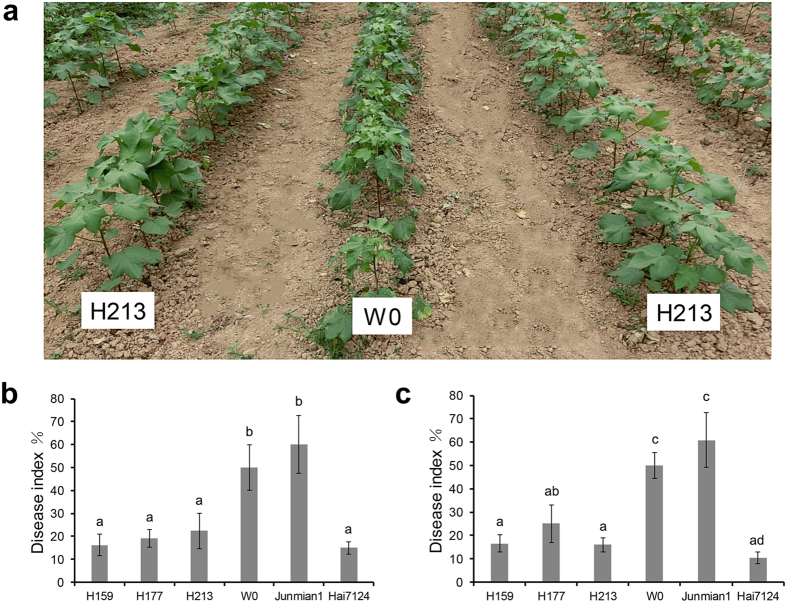
Resistance phenotypes of independent homozygous transgenic cotton lines. (**a**) Resistance phenotypes of independent transgenic cotton line H213 in *F. oxysporum*-inoculated field in Henan province, China in 2014. (**b**) The DIs of the transgenic and parent W0 plants induced by *F. oxysporum* isolate Fnj1 in greenhouse conditions. At least 30 plants were used for each experiment. (**c**) Severity of Fusarium wilt in *Hcm1*-transformed and parent W0 plants in the nursery. H159, H177, and H213 were the transgenic lines. Junmian 1 and Hai7124 were used as the susceptible and resistant controls. At least 15 plants were used for each experiment. Average values and standard errors were calculated from four independent experiments. The letters in (**a**,**c**) indicate significant differences at P ≤ 0.01 according to a randomization one-way ANOVA test.

*Hcm1* homozygous plants from three transgenic cotton lines, H159, H177, and H213, were first assessed in greenhouse bioassays for *F. oxysporum* resistance. In this bioassay, the progression of Fusarium wilt disease in the transgenic lines was compared to three control lines: the parent W0, a susceptible variety of Junmian 1, and a less susceptible variety, Hai7124. After 7 weeks, the disease progression in *Hcm1*-transformed lines and Hai7124 was statistically lower than in the parent W0 and the susceptible variety, Junmian 1. The susceptible variety, Junmian 1, had the highest disease index (DI) and the less susceptible variety, Hai7124, had the lowest DI. The three transgenic lines showed lower DIs compared to that of the parent W0 (Fig. 2b), implying that *Hcm1* improved the cotton’s resistance to Fusarium wilt caused by *F. oxysporum* in greenhouse conditions. The assessment of *Hcm1*-transformed lines’ resistance to Fusarium wilt under field conditions took place in Shangqiu city, Henan province, China, during the 2014 cotton-growing season. Seeds from *Hcm1*-transformed lines, parent W0, a susceptible variety, Junmian 1, and a less susceptible variety, Hai7124, were planted in a field where Fusarium wilt occurred heavily historically. The DI was investigated on June 24, 2014 in Henan according to historical peak incidences. These results showed that the DIs of the three transgenic lines were reduced to 66.77%, 49.83%, and 67.99% compared to the parent W0 (Fig. 2a,c), indicating that expressing *Hcm1* in cotton conferred resistance to Fusarium wilt in a field condition.

### Resistance of *Hcm1-*transformed cotton plants to Verticillium wilt

**Figure 3:**
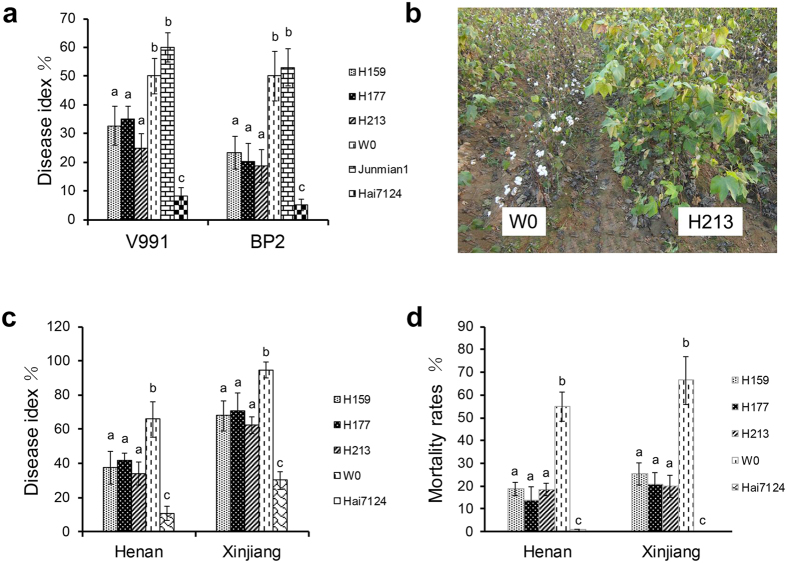
*Hcm1* transgenic lines improved resistance to Verticillium wilt. (**a**) The DIs of the transgenic and parent W0 cotton plants induced by defoliating *V. dahliae* strain V991 and non-defoliating *V. dahliae* strain BP2 in greenhouse conditions. H159, H177, and H213 were the transgenic lines. Junmian 1 and Hai7124 were used as susceptible and resistant controls. At least 45 plants were used for each experiment. Average values and standard errors were calculated from four independent experiments. (**b**) Resistance phenotype of the independent transgenic cotton line H213 in Henan province, China in 2014. (**c**,**d**) The DIs and mortality rates of *Hcm1*-transformed and parent W0 plants in a field with a history of a high incidence of Verticillium wilt. The transgenic lines, parent W0, and the resistance variety *G. barbadense* cv. Hai7124 were grown at a farm in Henan and Xinjiang province, China in the 2014 cotton-growing season. At least 15 plants were used for each experiment. Average values and standard errors were calculated from four independent experiments. The letters in (**a**,**c**,**d**) indicate significant differences at P ≤ 0.01 according to a randomization one-way ANOVA test.

Isolates of *V. dahliae* can be characterized as defoliating or non-defoliating pathotypes based on symptoms expressed in cotton plants with the disease^9^. The defoliating *V. dahliae* isolate V991 and non-defoliating *V. dahliae* isolate BP2 were used to assess the resistance of *Hcm1-*transformed cotton in greenhouse conditions. Foliar damage and vascular discoloration was observed in parent W0 plants at 10 and 15 days after inoculation with V991 and BP2, respectively. With the outbreak of the disease, the *Hcm1*-transformed plants had only a small number of chlorotic and necrotic spots and there was almost no plant death, whereas the parent W0 and susceptible variety, Junmian 1, plants showed common large chlorotic and necrotic areas in their leaves, and some plants eventually died (Fig. S1). The results showed that the DIs of the three transgenic lines were significantly lower than that of the parent W0 after inoculation with V991 and BP2, revealing that *Hcm1* improved cotton tolerance to defoliating and non-defoliating *V. dahliae* in greenhouse conditions (Fig. 3a).

Field trials to assess the performance of the transgenic lines against Verticillium wilt were conducted in Henan and Xinjiang provinces, in China during the 2014 cotton-growing season. Disease surveys were conducted on September 5, 2014 in Henan province and August 27, 2014 in Xinjiang province, since the peak incidence of Verticillium wilt in the field generally occurs in early August to mid-September in China. The DIs of the three transgenic lines decreased from 36.95% to 58.15% and from 25.33% to 34.03% compared to parent W0 (Fig. 3b,c) in Henan and Xinjiang provinces, respectively. The mortality rates of *Hcm1*-transformed lines decreased from 65.70% to 74.93% and 61.72% to 70.25% in Henan and Xinjiang, respectively (Fig. 3d). However, the resistance of transgenic lines to Verticillium wilt in Xinjiang was weaker than that in Henan. The reason may be the differences in climate, geographical conditions, or physiologic races in soils between the two provinces. In addition to the DI, the agronomic performance of the transgenic lines, including the height, lint percentage, number of fruit branches, single boll weight, and lint yield, was significantly higher than that of the parent W0 (Table 2). The lower DIs and mortality rates, and the better agronomic performance of *Hcm1*-transformed lines demonstrated that *Hcm1* conferred cotton resistance to Verticillium wilt, and improved its agronomic traits in the disease nurseries.

### ROS burst occurred and the PR genes were activated in *Hcm1*-transformed plants after inoculation with *V. dahliae*

**Figure 4:**
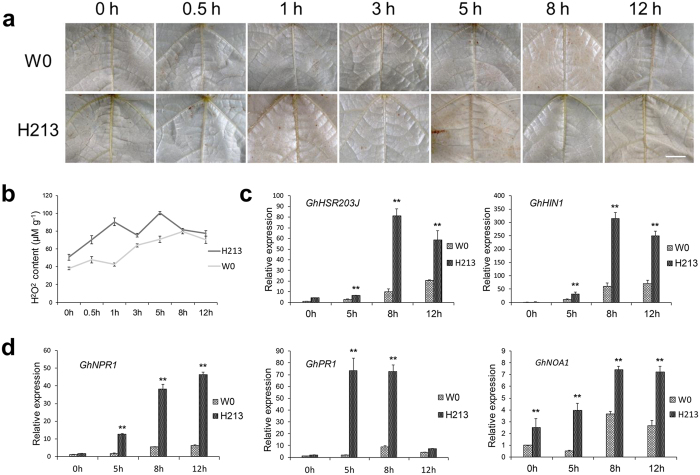
ROS burst in leaves of transgenic line H213 dipped in a conidial suspension of *V. dahliae*. (**a**) *In situ* observation of ROS in cotton leaves dipped in a conidial suspension of *V. dahliae* with DAB staining. The strong, brown precipitate was observed in leaves of parent W0 plants 8 hr after inoculation with *V. dahliae* and in leaves of transgenic line H213 plants 1 hr or 5 hr after inoculation with *V. dahliae*. At least six independent leaves were used for this experiment. A stereomicroscope (Olympus, DP72, Japan) was used for photographing the leaves under white light. Scale bars = 5mm. (**b**) H_2_O_2_ content (μM/g fresh weight) in leaves of transgenic line H213 and parent W0 plants dipped in a conidial suspension of *V. dahliae*. Error bars represent ± SE, n = 6. (**c**,**d**) The PR genes were activated in transgenic line H213 after inoculation with *V. dahliae*. Three biological replicates were used for each reaction with three technical replicates each. Mean values and standard errors were calculated from three biological replicates (**P ≤ 0.01, by student’s *t* test).

An ROS burst occurred when leaves of *hpa1*_*Xoo*_-transformed plants were inoculated with *V. dahliae*^14^. In the leaves of *Hcm1*-transformed line H213 and the parent W0 plants, a reddish-brown precipitate was observed after inoculation with *V. dahliae*, as detected by 3, 3′-diaminobenzidine tetrahydrochloride (DAB)^37^,^38^. However, the DAB staining in leaves of H213 plants was markedly different from that in parent W0 plants (Fig. 4a). In order to accurately observe this difference, H_2_O_2_ content was measured. The basal H_2_O_2_ content was higher in the leaves of transgenic H213 plants than in leaves of the parent W0 plants prior to inoculation. After inoculation with *V. dahliae*, H_2_O_2_ content gradually increased in leaves of the parent W0 plants, and peaked at 8 hours (hr), while in H213 leaves, H_2_O_2_ content was increased dramatically and appeared in two peaks at 1 hr and 5 hr (Fig. 4b). These results showed that the *Hcm1* transgenic lines displayed an ROS burst in response to biotic stress.

Harpins can activate the PR genes in plants^14^,^22^. *GhHSR203J* and *GhHIN1*, which are considered as marker genes for HR^20^,^21^, were up-regulated in the *Hcm1*-transformed line H213 and the parent W0 (Fig. 4c). *NPR1*, which is a key transcriptional regulator in plant defense responses involving multiple signaling pathways^39^, was up-regulated in transgenic line H213 after inoculation with *V. dahliae*. The marker genes of salicylic acid (SA) and nitric oxide (NO) signaling pathways^22^,^40^, *GhPR1* and *GhNOA1*, were also significantly up-regulated in the transgenic line H213 compared to the parent W0 (Fig. 4d), showing that the PR genes were activated in *Hcm1*-transformed plants in response to biotic stress.

### A microscopic hypersensitive response (micro-HR) was observed in transgenic line H213 after leaf and root inoculation with *V. dahlia*

**Figure 5:**
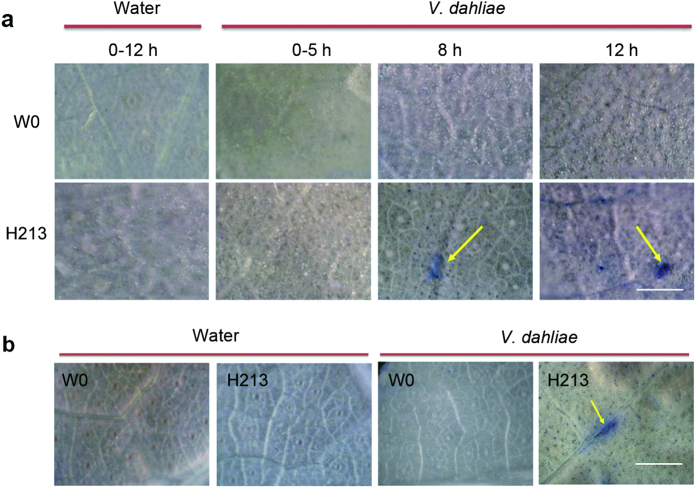
Micro-HR in leaves of *Hcm1*-transformed H213 plants after inoculation with *V. dahliae*. (**a**) Trypan blue staining of leaves from *Hcm1*-transformed H213 and parent W0 plants after inoculation with a conidial suspension of *V. dahliae*. No blue-violet coloration (representative of micro-HR) was observed 0–12 hr after inoculation with sterile water or a conidial suspension of *V. dahliae* in leaves of parent W0 plants. The leaves of transgenic line H213 showed no blue-violet coloration 0–12 hr after inoculation with sterile water or 0–5 hr after inoculation with a conidial suspension of *V. dahliae.* The blue-violet coloration (5–15 lesions per leaf; indicated by the yellow arrow) was observed in all leaves (≥2 leaves per plant) collected from transgenic plants infected with *V. dahliae* 8 or 12 hr post inoculation with *V. dahliae*. (**b**) Trypan blue staining of leaves from *Hcm1*-transformed H213 and parent W0 plants 15 days after root inoculation with *V. dahliae*. Occurrence of micro-HR (5–10 lesions per leaf, blue-violet coloration, indicated by yellow arrow) in leaves of transgenic line H213 plants but not parent W0. Root inoculation with sterile water as a control. A stereomicroscope (Olympus, DP72, Japan) was used for photographing the leaves under white light. Scale bars = 500 μm.

Harpins can induce an HR in tobacco following infiltration of leaf panels^2^. No visible HR was observed in cotton expressing *Hpa1*_*Xoo*_, but a micro-HR was detected in plants after inoculation with *V. dahliae*^14^. Leaves from transgenic line H213 and parent W0 plants 0–12 hr after inoculation with *V. dahliae* conidia suspension were stained with trypan blue, which selectively stains dead or dying cells^41^. Leaves inoculated with sterile water were used as a control. No trypan blue-stained cells were observed in leaves from H213 or W0 plants inoculated with water, or in parent W0 plants inoculated with *V. dahliae* (Fig. 5a). However, in leaves from H213 plants, trypan blue-stained cells representing a micro-HR were observed by stereoscope at 8 hr and 12 hr after inoculation with *V. dahliae* (Fig. 5a). Leaves from H213 and W0 plants after root inoculation with *V. dahliae* conidia suspension in greenhouse conditions were also used for micro-HR detection. Leaves inoculated with sterile water were used as a control. Trypan blue-stained cells were observed in leaves from H213 plants inoculated with *V. dahliae* but not in leaves from parent W0 plants inoculated with sterile water or *V. dahliae* or in H213 plants inoculated with sterile water, revealing that micro-HR occurred in *Hcm1*-transformed plants in response to biotic stress (Fig. 5b). These data indicate that a micro-HR occurred when the *Hcm1*-transformed plants suffered biotic stress.

### *Hcm1* effectively inhibits the spread of *V. dahliae* in cotton

**Figure 6:**
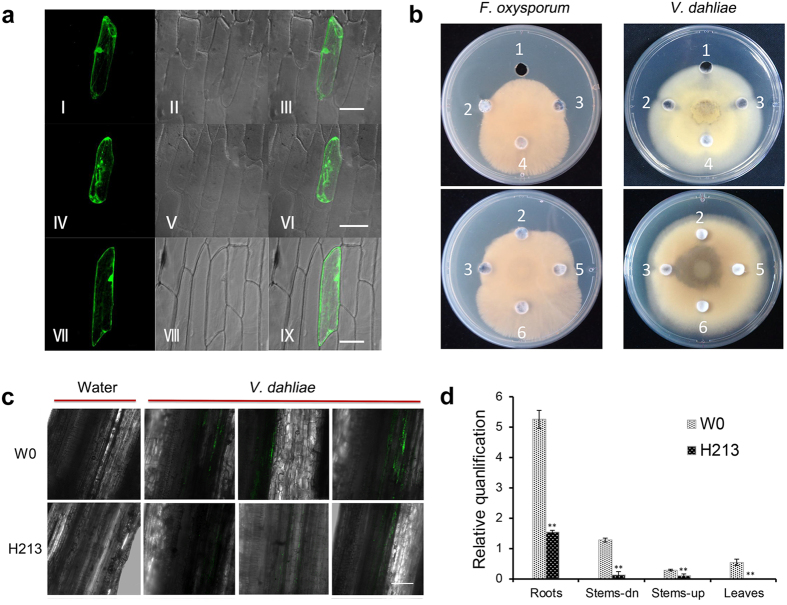
Expression of *Hcm1* inhibited the spread of fungal spores in cotton. (**a**) Localization of the 35S::*Hcm1*::GFP fusion in onion epidermal cells. (I–III) Localization of 35S::*Hcm1*::GFP in onion epidermal cells. (IV–VI) The cell wall and membrane were separated by treatment with 20% sucrose for 15 min. (VII–IX) 35S::GFP in onion epidermal cells (positive control). (I, IV, and VII) Onion cell under excitation at 488nm. (II, V and VIII) Onion cell under bright field. (III, VI and IX) GFP in the onion cell of overlayed images. Scale bars = 50 μm. (**b**) Antimicrobial activities of CFEPs and transgenic *Hcm1* protein against *F. oxysporum* Fnj1 and *V. dahliae* V991 on PDA plates. 1–6: 50 μg/ml Carbendazim, 1 mg/ml CFEPs of *Hcm1*, 1 mg/ml *Hcm1*-transformed protein, 1 mg/ml CFVPs, 200 μg/ml *Hcm1*-transformed protein, and 1 mg/ml parent W0 protein. (**c**) *In situ* observation of *V. dahliae* in rhizome connections of transgenic line H213 and parent W0 plants 15 days after inoculation with *V. dahliae* harboring the GFP gene. At least 15 plants of transgenic lines H213 and parent W0 plants were used in this study. The freehand sections were obtained and checked by laser scanning confocal microscopy. Scale bar = 200 μm. (**d**) Detection of the *V. dahliae* biomass in transgenic line H213 and parent W0 plants using qRT-PCR. DNA was extracted from roots, the lower half of stems (stem-dn), the upper half of stems (stem-up) and the first leaves of plants 15 days after inoculation with *V. dahliae*. The relative average fungal biomass is shown with standard errors. Asterisks indicate significant differences when compared with colonization of the parent W0 plants (**P ≤ 0.01, by student’s *t* test).

Cecropin A-melittin can normally inhibit pathogens infection when the Hcm1 protein exists in the plasma membrane. Moreover, recent studies have shown that host targets of harpins may be present in the plasma membrane^20^,^42^,^43^. Our unpublished data suggest that *Hpa1*_*Xoc*_is located in the plasma membrane and nuclear membrane of plant cells. Therefore, whether the Hcm1 protein exists in the plasma membrane is very important for the function of *Hpa1*_*Xoc*_ and cecropin A-melittin from *Hcm1*. We fused the *Hcm1* coding region in frame with the N-terminus of GFP coding region under the control of the CaMV35S promoter to examine the subcellular localization of *Hcm1*. Onion epidermal cells were separately transformed with either the 35S::*Hcm1*::GFP fusion or the 35S::GFP plasmid control by particle bombardment. GFP-specific fluorescence was found in the cell membrane and other parts of cells transformed with the 35S::*Hcm1*::GFP fusion (Fig. 6a I–III). When the cell wall and cell membrane were separated by treatment with 20% sucrose for 15 min, GFP fluorescence was observed in the membrane but not in the cell wall (Fig. 6a IV–VI). GFP fluorescence was detected throughout control cells transformed with the 35S::GFP plasmid (Fig. 6a VII–IX). These results indicate that *Hcm1* is present in the cell membrane when *Hcm1* is expressed in plant cells.

The Hcm1 protein shows broad antimicrobial activity *in vitro*^22^. The ability of crude cell-free elicitor preparations (CFEPs) of *Hcm1* and the *Hcm1*-transformed proteins, which were extracted from prokaryotic expression and the leaves of transgenic line H213 plants, respectively, to inhibit the growth of *V. dahliae* and *F. oxysporum* on potato dextrose agar (PDA) and complete medium (CM) plates was tested. Carbendazim, CFEPs and *Hcm1*-transformed proteins caused an obvious inhibition halo on PDA and CM plates inoculated with *F. oxysporum*. A 5-fold dilution of *Hcm1*-transformed proteins also inhibited the growth of *F. oxysporum* compared to controls (Fig. 6b and Fig. S2). Carbendazim, CFEPs, and *Hcm1*-transformed proteins also inhibited the mycelial growth of *V. dahliae* on PDA and CM plates, while a 5-fold dilution of *Hcm1*-transformed protein had no effect on the growth of *V. dahliae* (Fig. 6b and Fig. S2).

In order to verify the antimicrobial activity of *Hcm1* against *V. dahliae in vivo*, the strength of green fluorescence was observed in cotton plants inoculated with a *V. dahliae* strain V991 harboring the GFP gene , since *V. dahliae* harboring the GFP gene emits fluorescence in cotton tissues^44^. 15 days after inoculation with *V. dahliae,* the green fluorescent signal was observed in the leaves of parent W0 plants but not *Hcm1*-transformed plants (Fig. S3). Although the green fluorescent signal was observed in the rhizome connections of *Hcm1*-transformed and parent W0 plants, the green fluorescent signal in *Hcm1*-transformed plants was significantly weaker than in parent W0 plants (Fig. 6c). In addition to observing the fluorescent signal, the biomass of *V. dahliae* strain V991 in cotton plants was measured with qRT-PCR. Determination of the *V. dahliae* strain biomass showed significant differences between *V. dahliae*-inoculated *Hcm1*-transformed and parent W0 plants. The biomass of *V. dahliae* strain V991 in the roots, stems, and leaves of *Hcm1*-transformed plants was significantly lower than in parent W0 plants (Fig. 6d). These results revealed that the expression of *Hcm1* reduced the biomass of *V. dahliae* in cotton plants. *Hcm1*, therefore, effectively inhibited the spread of *V. dahliae* in cotton.

## Discussion

### *Hcm1* was effective at controlling Fusarium wilt and Verticillium wilt

Previous studies have shown that harpin, applied as a foliar spray or expressed in plants, confers resistance to pathogens^14^,^24^,^45^,^46^. The antimicrobial proteins, cecropin A-melittin, also effectively confer plants with a resistance to a broad spectrum of pathogens^33^,^34^,^35^. Resistance to tobacco mosaic virus, bacterial *Ralstonia solanacearum*, and fungal *Magnaporthe oryzae* infections can be improved by spraying Hcm1 protein on plants prior to inoculation with plant pathogens^22^. In the present study, *Hcm1* was transformed into a susceptible cotton variety, W0. In greenhouse conditions, the *Hcm1*-transformed cotton lines were resistant to disease caused not only by the defoliating and non-defoliating isolates of *V. dahliae*, but also *F. oxysporum*. In the *V. dahliae* and *F. oxysporum*-inoculated field, the *Hcm1*-transformed plants showed lower DIs and mortality rates compared to parent W0 plants (Figs 2 and 3). The lint yields, which are regarded as the most important agronomic measurement of cultivar performance, of *Hcm1*-transformed and parent W0 plants were not significantly different when the plants were grown in non-infected soil in field conditions (Table 1). When planted in *V. dahliae*-infected soil, the lint yields of *Hcm1*-transformed plants were 20–77% higher in Henan province and 27–73% in Xinjiang province than parent W0 plants (Table 2). The results indicate that *Hcm1* is effective at controlling Fusarium and Verticillium wilts.

### *Hcm1* led to an ROS primed state for plant defense activation in cotton

Higher plants are capable of inducing some stress “memory” or “stress imprinting” as a primer induced by the first exposure to a stress that leads to enhanced resistance to a later stress^47^,^48^. ROS, as signaling molecules, play a key role in such priming events^49^. The harpin protein, *Hpa1*_*Xoo*_, which is isolated from *Xanthomonas oryzae*, confers resistance to Verticillium wilt by activating a priming mechanism in cotton. A rapid burst of ROS was observed in *Hpa1*_*Xoo*_-transformed plants after inoculation with *V. dahliae*^14^. Our previous study showed that Hcm1 protein possesses the same characteristics as the harpin protein Hap1_Xoc_^22^. In addition, *Hcm1,* like *Hpa1*_*Xoc*_, is located in the plasma membrane (Fig. 6a), which may be necessary for the function of harpins^20^,^42^,^43^. In *Hcm1*-transformed plants, H_2_O_2_ content was slightly higher than in parent W0 plants and a ROS burst occurred after inoculation with *V. dahliae* (Fig. 4a,b). SA and ROS interplay in the transcriptional control of defense gene expression and play an important role in the disease resistance of plants^50^. On infection of hexanoic acid-treated plants, hexanoic acid activates the SA pathway as part of the priming mechanism^51^. Recent studies show that NO is another key signaling molecule involved in the induction of protection against biotic and abiotic factors through a complex network^40^. Harpins can activate the expression of PR genes such as *NPR1, PR1-a* (an SA marker)^21^,^22^,^23^, and *HSR203J* and *HIN1* (HR markers)^21^,^41^,^48^,^52^. The up-regulation of *GhNPR1, GhPR1*, and *GhNOA1* in response to pathogen infection was observed in *Hcm1*-transformed plants, revealing that the signaling pathways of SA and NO were activated (Fig. 4d). These results indicate that *Hcm1* may lead to a primed state in cotton, and result in a faster and stronger induction of basal resistance mechanisms upon pathogenic attacks, since the priming was accompanied by an ROS burst, SA accumulation, and the induction of PR genes^14^,^53^. All harpins reported thus far, except *XopA*^54^ and truncated *HrpZ1*^55^, can induce an HR in plants. *Hcm1*, which contains a harpin, also induces an HR *in planta*^14^,^22^. In *Hcm1*-transformed plants, the HR marker genes, *HSR203J* and *HIN1*, were activated (Fig. 4c) and a micro-HR was observed after inoculation with *V. dahliae* (Fig. 5). Miao *et al.*^14^ suggested that such a micro-HR may augment the response to infections caused by fungal pathogens.

### *Hcm1* may provide antimicrobial properties in cotton

Cecropin A-melittin of Hcm1 protein has been shown to effectively inhibit the growth of a variety of pathogens *in vitro*^22^. The Hcm1 protein extracted from prokaryotic expression or transgenic line H213 plants inhibited the growth of *V. dahliae* and *F. oxysporum in vitro* (Fig. 6b). The Hcm1 protein was located in the plasma membrane of plant cells (Fig. 6a), which may be help cecropin A-melittin to inhibit pathogens. In *Hcm1*-transformed plants, the biomass of *V. dahliae* was markedly lower than in parent W0 plants, as determined by qRT-PCR analysis and by observing the fluorescent signal strength of *V. dahliae* harboring the GFP gene (Fig. 6d). These results showed that the spread of *V. dahliae* was effectively hindered. A synthetic chimera of cecropin A and melittin CAPs with antimicrobial properties, MsrA1, effectively restricts *Alternaria brassicae* and *Sclerotinia sclerotiorum* infection in transgenic *Brassica juncea* and *Solanum tuberosum* plants^34^,^56^. The novel cecropin A-melittin hybrid peptide, CEMA, which has strong antimicrobial activity *in vitro*, confers resistance against *Fusarium solani* in transgenic tobacco^57^. Our previous studies have shown that the disease resistance conferred by Hcm1 protein is more effective than that of the Hpa1_Xoc_ protein when Hcm1 or Hpa1_Xoc_ proteins are sprayed onto plants^22^. These results indicate that the cecropin A-melittin of the Hcm1 protein may also contribute to resistance against Fusarium and Verticillium wilts in transgenic cotton. The improved resistance to Fusarium and Verticillium wilts in cotton plants conferred by the Hcm1 protein may be a joint action of the Hpa1_Xoc_ protein and cecropin A-melittin.

In conclusion, these results lead us to suppose that the Hpa1_Xoc_ protein from Hcm1 activates a ROS priming mechanism in transgenic plants in response to *V. dahliae* and *F. oxysporum* infection, and cecropin A-melittin from Hcm1, which is located in the plasma membrane, simultaneously inhibits the spread of *V. dahliae* and *F. oxysporum* to confer resistance to both Verticillium and Fusarium wilts in cotton.

### Future potential for fusion protein

Verticillium wilts are among the most devastating fungal diseases worldwide and affect hundreds of different plant species including high value agricultural crops^58^. Economic losses of 50% or higher commonly occur in high value crops, including cotton^59^, lettuce^60^, olive^61^, and potato^62^. *F. oxysporum* was also described as an important fungal pathogen in a survey of plant pathologists, based on its economic and scientific importance^63^. Plant genetic engineering has been made possible thanks to extensive research conducted during the last three decades. Several studies have reported the control of *F. oxysporum* and *V. dahliae* infection by transgenic approaches^6^,^7^,^8^,^9^,^10^,^11^,^12^,^13^,^14^. *Hcm1*, a novel protein that induces plant defense responses and directly inhibits microbial growth, could improve cotton resistance to Verticillium and Fusarium wilts and offer a considerable yield advantage. Our previous^22^ and present studies showed that *Hcm1* confers resistance to multiple pathogens either by spraying on plants or expressing in plants, indicating that fusion proteins like Hcm1 could be widely applied to other crops in future to improve defense against plant diseases and improve crop yields.

## Materials and Methods

### Plant materials and *V. dahliae* and *F. oxysporum* culture

The transgenic cotton plants, as well as their parent W0, the resistant control for Verticillium wilt and Fusarium wilt, *Gossypium barbadense* (*G. barbadense*) cv. Hai7124, and the susceptible control for Verticillium and Fusarium wilts, *G. hirsutum* cv. Junmian 1, were grown in the green house facility in Nanjing Agricultural University in China. The growing conditions were: a constant temperature of 28 °C, a relative humidity of 70%, and a 16 hr photoperiod. Highly aggressive defoliating *V. dahliae* isolate V991 was stored in our laboratory and non-defoliating *V. dahliae* isolate BP2 was provided by the Institute of Plant Protection, Jiangsu Academy of Agricultural Sciences. *V. dahliae* V991 harboring the GFP gene was provided by the Biotechnology Institute, Jiangsu Academy of Agricultural Sciences. *V. dahliae* was maintained on PDA at 25 °C for 5 days, inoculated into Czapek’s medium^64^, and then shocked at 25 °C for 5 days. *F. oxysporum* isolate Fnj1, which was provided by Institute of Plant Protection, Jiangsu Academy of Agricultural Sciences, was maintained on PDA at 24 °C for 3–5 days, inoculated into Czapek’s medium and shocked at 25 °C for 3 days. Before inoculation, the conidia were counted and the conidia suspension was adjusted to the required density with distilled water.

### Cotton transformation and transgenic plant selection

The recombinant binary vector pBI35S-*Hcm1*-*NPT*II, which contained aneomycin phosphotransferase II (*NPT*II) with a nopaline synthase (*Nos*) promoter and terminator, a CaMV35S promoter, an *Hcm1* insert, and a *Nos* terminator, was transformed into W0 plants. *Agrobacterium*-mediated cotton transformation was performed as described previously^65^. After induction, differentiation, and plantlet regeneration, the plantlets were grafted onto rootstocks and grown in a greenhouse. The homozygosity of transgenic plants were determined by analyzing the segregation ratio of the kanamycin selection marker and by PCR analysis. Kanamycin resistance tests, PCR analysis, Southern blots, Western blots, and resistance to Verticillium wilt were used to screen T_1_ to T_6_ progeny for *Hcm1*-transformed cotton lines.

### Southern and Western blots analysis

The method of Southern blots analysis was conducted as described by Lv *et al.*^66^. 20 μg gDNA from the leaves of *Hcm1*-transformed and parent W0 plants was digested with *Eco*RI. Probes were prepared from purified PCR products of the *NPT*II coding region. The labeling of probes, prehybridization, hybridization, and detection were performed according to the protocol of the DIG High Prime DNA Labeling and Detection Starter Kit I (Roche Applied Science, Mannheim, Germany).

Total protein was extracted from the leaves of *Hcm1*-transformed and parent W0 plants according to the manufacturer’s instructions of the Plant Protein Extraction Kit (CWBIO, Beijing, China). Protein concentration was measured according to the manufacturer’s instructions for the BCA Protein Assay Kit (Solarbio, Beijing, China). Total proteins were separated by 12% sodium dodecyl sulfate-polyacrylamide gel electrophoresis (SDS-PAGE) and then transferred onto a polyvinylidene fluoride (PVDF) membrane (Roche Applied Science, Mannheim, Germany). The membrane was blotted with a polyclonal antibody developed against *Hcm1* and a goat anti-rabbit IgG-HRP antibody (Sino-American Biotech, Luoyang, China). The color was developed using DAB.

### qRT-PCR

Total RNA from leaves, stems and roots of *Hcm1*-transformed and parent W0 plants was isolated using the CTAB method^67^, and 2 μg of total RNA was used for reverse transcription. *EF-1α* (Table S1) from cotton was used as an internal control for normalization of the different cDNA samples. The PR genes in cotton are listed in Table S2. The primer sequences for PR genes are shown in Table S1. PCR was performed using the real-time PCR system (Bio-Rad) along with SYBR Green PCR Master Mix (Applied Biosystems). Each PCR was repeated three times, and the data were evaluated using the comparative cycle threshold method described by Livak and Schmittgen^68^.

### Evaluation of resistance to Verticillium wilt and Fusarium wilt in greenhouse conditions

For the determination of Verticillium wilt resistance, after surface disinfection for 5 min with a 5% solution of sodium hypochlorite, cotton seeds were sown in a potting mixture (peat:vermiculite, 1:1, v/v). Thirty 18-day-old cotton seedlings were inoculated with defoliating *V. dahliae* isolate V991 and non-defoliating *V. dahliae* isolate BP2 by soil drenching with 20 ml conidial suspension (5 × 10^6^ conidia/ml) for each pot (250 ml), and were grown under the following conditions: 12 hr of light at 25 °C and 70–90% relative humidity. Plants in the control group received same amount of sterile water. The DI was measured after two weeks in a greenhouse. After inoculated with non-defoliating *V. dahliae* isolate BP2, foliar damage was evaluated by rating the symptom on the cotyledon and leaf of inoculated plant according to the following disease grades: 0 = healthy plants, no fungal infection, 1 = 25% of the leaves showing yellowing or abnormal yellow spots, 2 = 25 to 50% of the leaves showing yellow spots, 3 = 50 to 75% of the leaves showing brown spots and curled leaf edges, and 4 = >75% of the leaves showing yellow spots or irregular yellow spots between the main vein of leaves. After inoculated with defoliating *V. dahliae* isolate V991, foliar damage was evaluated by rating the symptom on the cotyledon and leaf of inoculated plant according to the following disease grades: 0 = healthy plant, 1 = yellowing or necrosis of 1–2 cotyledons, 2 = yellowing or necrosis of 1 true leaf, 3 = more than 2 wilted or necrotic leaves, 4 = no leaf left or dead plant.

For the determination of *F. oxysporum* resistance, a spore suspension of Fnj1 was added to sterilized strain bags containing grains of wheat. The grains of wheat were removed and dried after 20 days, before being mixed with mould and sand (1:1, v/v) at a ratio of 3% and encased an aluminum skin frame (45 cm × 33 cm × 16 cm). After surface disinfection, cotton seeds were sown in the aluminum skin frame and grown with 12 hr of light, at 25 °C and 70% - 90% relative humidity. The DI was measured after seven weeks in a greenhouse. After inoculated with *F. oxysporum* isolate Fnj1, foliar damage was evaluated by rating the symptom on the cotyledon and leaf of inoculated plant according to the following disease grades: 0 = healthy plants, no fungal infection, 1 = 25% of the leaves showing yellowing or wilting, 2 = 25 to 50% of the leaves showing yellowing or wilting, leaf veins showing yellow, 3 = 50 to 75% of the leaves showing yellowing or wilting, cotton plants dwarf or wilting, and 4 = >75% of the leaves showing yellowing or wilting, cotton plants dead.

The disease index was calculated as according to the formula: DI = [∑disease grades × number of infected plants)/(total checked plants × 4)] × 100^9^,^11^. At least thirty individual plants per line were subjected to resistant analysis and each experiment was repeated four times.

### Evaluation of resistance to Verticillium and Fusarium wilts in field conditions

For resistance assessment, the transgenic lines, the parental line W0, and the resistant variety, Hai7124 were grown in the Verticillium wilt nurseries in the Henan and Xinjiang provinces in China. To assess their agronomic performance, the transgenic lines and the parental line W0 were also planted at a farm without diseased soil in Jiangsu Province, China, in 2014. All seeds were treated with an insecticide (Pymetrozine, J&K, San Diego, USA) prior to planting to protect against thrip and aphid damage, and some seeds were also treated with dynasty fungicide to control damping off diseases. In the Verticillium wilt nurseries of Henan province and Xinjiang province, the seed densities were 5 seeds m^–1^ and 25 seeds m^–1^, respectively. Each treatment was replicated four times, and the replicates were arranged in a randomized complete block design. The DI was calculated using the above formula. At the completion of the trial, fifteen successive plants were chosen and tagged in each plot. The height of plants, number of fruit branches, and boll number per plant were investigated. Twenty-five bolls were manually harvested in each plot to investigate the weight of boll and lint percentage. Total cotton yield was assessed by hand picking all harvestable bolls.

### Subcellular localization of *Hcm1*

The full length *Hcm1* coding region was inserted between the cauliflower mosaic virus 35S promoter and terminator sequences in the pJIT166-GFP vector by PCR with linker primers (Table S1) that contained *Hind*III and *Xba*I sites. All plasmid constructs were confirmed by sequencing. The 35S::GFP control and 35S::*Hcm1*::GFP vectors were transiently expressed in onion epidermal cells using a biolistic particle delivery system (PDS-1000 Bio-Rad, USA). The subcellular localization of the 35S::*Hcm1*::GFP fusion protein was observed with a confocal laser scanning microscope (LSM 510, Zeiss, Germany).

### Observation of ROS burst and quantification of H_2_O_2_ in cotton leaves

The third cotton leaves that had no visible wounds were selected when the plants were at the 4 leaf stage and the leaf surfaces were smeared with the conidial suspension of *V. dahliae* (1–3 × 10^7^ ml) and incubated for 0, 0.5, 1, 3, 5, 8, or 12 hr. To visualize the accumulation of H_2_O_2_, the fresh cotton leaves were collected and incubated in 1 mg/ml of DAB (pH 3.8) for 8 hr and then decolorized in 96% ethanol. The samples were examined with a stereo microscope lens (Olympus, DP72, Germany). The accumulation of H_2_O_2_ was visible as red-brown discoloration. The production of H_2_O_2_ in leaves was also measured with a commercial H_2_O_2_ detection kit (Nanjing Jiancheng Bioengineering Institute, Nanjing, China) using the method described by Jiang and Zhang^69^ and expressed as a percentage of fresh weight. Leaves dipped in sterile water were used as the negative control. Each experiment was repeated six times.

### Microscopic investigation of micro hypersensitive response

The third cotton leaves that had no visible wounds were selected and the leaf surfaces were smeared with the conidial suspension of *V. dahliae* (1–3 × 10^7^ ml). Leaves were collected at 0, 0.5, 1, 3, 5, 8, or 12 hr and stained with trypan blue using the method described by Lipka *et al.*^41^. In addition, roots of transgenic line H213 and parent W0 plants at the 2 to 3 leaf stage were inoculated with a conidial suspension of *V. dahliae* (5 × 10^6^/ml) according to the method described above. Leaves were collected 15 days after inoculation with *V. dahliae* and then stained with trypan blue. Stained leaf samples were observed under a Leica light microscope (Leica DMRB, Leica Microsystems, Germany) and photographed with a Leica DFC camera (DM2500-3HF-FL, Leica Microsystems, Germany). Leaves without any wounds or visible symptoms of the disease from 10 independent H213 plants were examined.

### Qualitative and quantitative detection of *V. dahliae* in cotton

Roots of transgenic line H213 and parent W0 plants at the 2 to 3 leaf stage were inoculated with a conidial suspension of *V. dahliae* harboring the GFP gene (5 × 10^6^/ml) according to the method described above. 15 days post-inoculation, the fluorescence signal strength at leaves and the rhizome connections were detected using a laser scanning confocal microscope (LSM 510, Zeiss, Germany). As the quantity of *V. dahliae* increases, the intensity of the fluorescent signal from GFP also increases. The roots, lower half of the stems, upper half of the stems, and first true leaves were also collected to use for DNA extraction. The internal transcribed spacer region of the ribosomal DNA was targeted to generate a 200 bp amplicon to measure the biomass of *V. dahliae*, using the fungus-specific primer W9500F^70^ and the *V. dahliae*-specific reverse primer W9500R^71^. *EF-1α* from cotton was used as an internal control for normalization of the different DNA samples. The average fungal biomass was determined using at least six *V. dahliae*-inoculated plants for each genotype, and quantified in plants as described by Ellendorff Ursula *et al.*^72^.

### Antimicrobial spectrum for *Hcm1*

CFEPs of *Hcm1* were obtained using prokaryotic expression technology according to the methods described by Che *et al.*^22^. *Hcm1*-transformed protein was also extracted from the leaves of *Hcm1*-transformed plants. 10 μl spore suspension of *V. dahliae* isolate V991 and *F. oxysporum* isolate Fnj1 was placed in the center of PDA or CM plates and then 5 mm diameter holes were made around the mycelial discs using a hole puncher. One hundred microliters of *Hcm1* protein was added to the holes on the PDA or CM plates. The plates were incubated at 28 °C for 3–5 days depending on the fungal growth rate. Carbendazim (50 μg ml^−1^) was used as the positive control for the fungi. The crude cell-free vector preparations (CFVPs) and the proteins from parent W0 plants were used as negative controls.

## Additional Information

**How to cite this article**: Zhang, Z. *et al.* Constitutive expression of a novel antimicrobial protein, *Hcm1*, confers resistance to both Verticillium and Fusarium wilts in cotton. *Sci. Rep.* **6**, 20773; doi: 10.1038/srep20773 (2016).

## Supplementary Material

Supplementary Information

## Figures and Tables

**Table 1:** The agronomic performance of *Hcm1*-transformed and non-transgenic parent W0 plants at a farm known not to harbor cotton fungal pathogens in Jiangsu province.

**Lines**	**Height(cm)**	**No. fruit branch per plant**	**Boll number per plant**	**boll weight (g)**	**Lint percent (%)**	**Seed cotton yield(kg)**
H159	101.9 ± 3.9	17.6 ± 0.5	19.5 ± 1.6	3.28 ± 0.38	37.1 ± 1.5	165.33 ± 20.9
H177	108.2 ± 7.6	18.7 ± 2.2	20.3 ± 1.4	3.35 ± 0.19	37.5 ± 2.2	170.01 ± 14.9
H213	112.3 ± 9.6	17.8 ± 1.2	22.6 ± 1.9	3.59 ± 0.39	39.3 ± 2.4	202.54 ± 39.2
W0	115.9 ± 8.5	17.7 ± 2.9	20.7 ± 2.6	3.57 ± 0.13	38.1 ± 1.1	185.71 ± 14.2

The cotton plants during growth development did not show any disease. Data represent the mean ± SE (n ≥15); similar results were obtained from four independent experiments.

**Table 2:** The agronomic performance of *Hcm1*-transformed and non-transgenic parent W0 plants in a field with a history of a high incidence of *V. dahliae*.

	**Lines**	**Height(cm)**	**No. fruit branch per plant**	**Boll number of per plant**	**Single boll weight (g)**	**Lint percent (%)**	**Seed cotton yield(kg)**
Henan	H159	94.9 ± 3.9^a^	7.1 ± 0.7	11.1 ± 1.6^a^	3.16 ± 0.21^a^	37.6 ± 1.2^a^	122.58 ± 27.4^a^
	H177	102.2 ± 7.6^b^	8.1 ± 1.5	12.4 ± 2.0^a^	3.66 ± 0.14^a^	39.3 ± 2.3^a^	158.92 ± 22.7^ab^
	H213	101.3 ± 9.6^b^	7.8 ± 0.6	13.6 ± 0.9^a^	3.72 ± 0.79^a^	38.5 ± 1.4^a^	177.29 ± 16.5^b^
	W0	86.3 ± 7.5^c^	6.5 ± 0.8	7.6 ± 1.1^b^	2.97 ± 0.39^b^	35.5 ± 2.4^b^	79.01 ± 14.8^c^
Xinjiang	H159	57.3 ± 2.9^a^	7.8 ± 0.4	4.8 ± 1.6^a^	3.70 ± 0.61^a^	43.9 ± 1.1^a^	316.3 ± 32.4^a^
	H177	62.5 ± 2.3^a^	7.8 ± 1.0	5.6 ± 0.6^a^	3.89 ± 0.45^ab^	44.0 ± 0.5^a^	390.1 ± 41.6^ab^
	H213	60.3 ± 1.9^a^	8.4 ± 1.1	5.8 ± 1.7^a^	4.14 ± 0.57^b^	44.3 ± 0.6^a^	432.3 ± 48.5^b^
	W0	48.5 ± 2.7^b^	7.6 ± 1.3	4.3 ± 0.8^b^	3.26 ± 0.32^c^	42.6 ± 0.9^b^	249.5 ± 33.8^c^

The transgenic lines, parent W0, and the resistance variety *G. barbadense* cv. Hai7124 were grown at a farm in Henan and Xjinjiang province, China in the 2014 cotton-growing season. Seeds were planted in a field with a history of a high incidence of Verticillium wilt caused by *V. dahliae*. Data represent the mean ± SE (n ≥ 15); similar results were obtained from four independent experiments. The letters indicate significant differences at P ≤ 0.05 according to a randomization one-way ANOVA test.
