# Supplementary material for: Constitutive expression of a novel antimicrobial protein, Hcm1, confers resistance to both Verticillium and Fusarium wilts in cotton
Source: Sci Rep. 2016 Feb 9;6:20773. doi: 10.1038/srep20773 (PMC4746735; doi:10.1038/srep20773)
Supplement: Supplementary Information [file srep20773-s1.doc]

**Title:**

**Constitutive expression of a novel antimicrobial protein, *Hcm1*, confers resistance to both *Verticillium* and *Fusarium* wilts in cotton**

**Journal name: *Scientific Reports***

**Zhiyuan Zhang^1^, Jun Zhao^1^, Lingyun Ding^1^, Lifang Zou^2^, Yurong Li^2^, Gongyou Chen^2*^, Tianzhen Zhang^1*^**

1. National Key Laboratory of Crop Genetics & Germplasm Enhancement, Cotton Research Institute, Nanjing Agricultural University, Nanjing 210095, P. R. China
2. School of Agriculture and Biology, Shanghai Jiao Tong University/Key Laboratory of Urban (South) by Ministry of Agriculture, Shanghai, China

******* Correspondence and requests for materials should be addressed to Tianzhen Zhang (cotton@njau.edu.cn).

**Supporting Information**

**Table S1.** The primers used in the research.

**Table S2.** PR genes in cotton.

**Figure S1.** Phenotype of *Hcm1*-tansformed plants and parent W0 plants at 2 weeks after inoculation with *V. dahliae* isolate V991 and 3 weeks after inoculation with *V. dahliae* isolate BP2.

**Figure S2.** Antimicrobial activities of CFEPs and transgenic protein of *Hcm1* tests against *F. oxysporum* Fnj1 and *V. dahliae* V991 on CM plates.

**Figure S3.** The distribution of the green fluorescent signal in first true leaves of transgenic line H213 and parent W0 plants 15 days after inoculation with *V. dahliae* harboring the GFP gene.

**Supporting Tables**

**Table S1** The primers used in the research

| **Primers** | **sequence(5'-3')** | **Purposes** |
| --- | --- | --- |
| *Hcm1*-pBI121-F | GAGTCTAGAATGAACTCTTTGAACACA | Vector construction for pBI121-*Hcm1* |
| *Hcm1*- pBI121-R | CTTCCCGGGCTAGAGACCCGTGGTGAG |
| *Hcm1*-F | TCACCCAGATGCTGATGAATA | qRT-PCR for *Hcm1* |
| *Hcm1*-R | GAAACTGCCACCAAAGTCG |
| *NPT*II-F | GAGGCTATTCGGCTATGACTG | *NPT*II gene-specific primers |
| *NPT*II-R | TAGAAGGCGATGCGCTGCGA |
| *Hcm1*-F | TAAGGGATGACGCACAATC | PCR for *Hcm1*-transformed plants |
| *Hcm1*-R | CGAAACTGCCACCAAAGT |
| *Hcm1*-GFP-F | CCCAAGCTTATGAACTCTTTGAACACACAAT | Vector construction for 35S::*Hcm1*::GFP fusion |
| *Hcm1*-GFP-R | GCTCTAGAGAGACCCGTGGTGAGCACC |
| *EF1α*-F | AGACCACCAAGTACTACTGCAC | internal standard primer for cotton |
| *EF1α*-R | CCACCAATCTTGTACACATCC |
| *V. dahliae*-F | AAAGTTTTAATGGTTCGCTAAGA | internal standard primer for *V. dahliae* |
| *V. dahliae*-R | CTTGGTCATTTAGAGGAAGTAA |
| *GhNPR1*-F | TCAGTTTAGACAAGCCCGAGAA | qRT-PCR for *GhNPR1* |
| *GhNPR1*-R | CGTATGACCCTCTTTCAGTAGCA |
| *GhPR1*-F | TGCTGTAAATATGTGGGTTAATGAG | qRT-PCR for *GhPR1* |
| *GhPR1*-R | GAAATTGCCTGGAGGAGAATAG |
| *GhNOA1*-F | GAGGATGCTGAAAGACCTGCTA | qRT-PCR for *GhNOA1* |
| *GhNOA1*-R | TCTCAACTGGCTTGGGTACATG |
| *GhHSR203J* -F | GTGATAGCTCAGGAGGGAACAT | qRT-PCR for *GhHSR203J* |
| *GhHSR203J* -R | CTAACTCGGACTTGCTTCGTTG |
| *GhHIN1*-F | GCTGATGAGACATCGGAGTTTA | qRT-PCR for *GhHIN1* |
| *GhHIN1*-R | CTACCATTCCCAGTGTTCAAAG |

**Table S2 The agronomic performance and yield of the transgenic and non-transgenic**

| Protein | Homologous protein | Accession No. | Homology |
| --- | --- | --- | --- |
| *GhNPR1* | *GhNPR1* | U76707 | 100 |
| *GhPR1* | *AtPR1* | NM_127025.2 | 66.5 |
| *GhNOA1* | *NtNOA1* | BAF93184.1 | 70.4 |
| *GhHSR203J* | *NtHSR203J* | X77136 | 70.3 |
| *GhHIN1* | *NtHIN1* | Y07563 | 60 |

**Supplementary Figures**

**
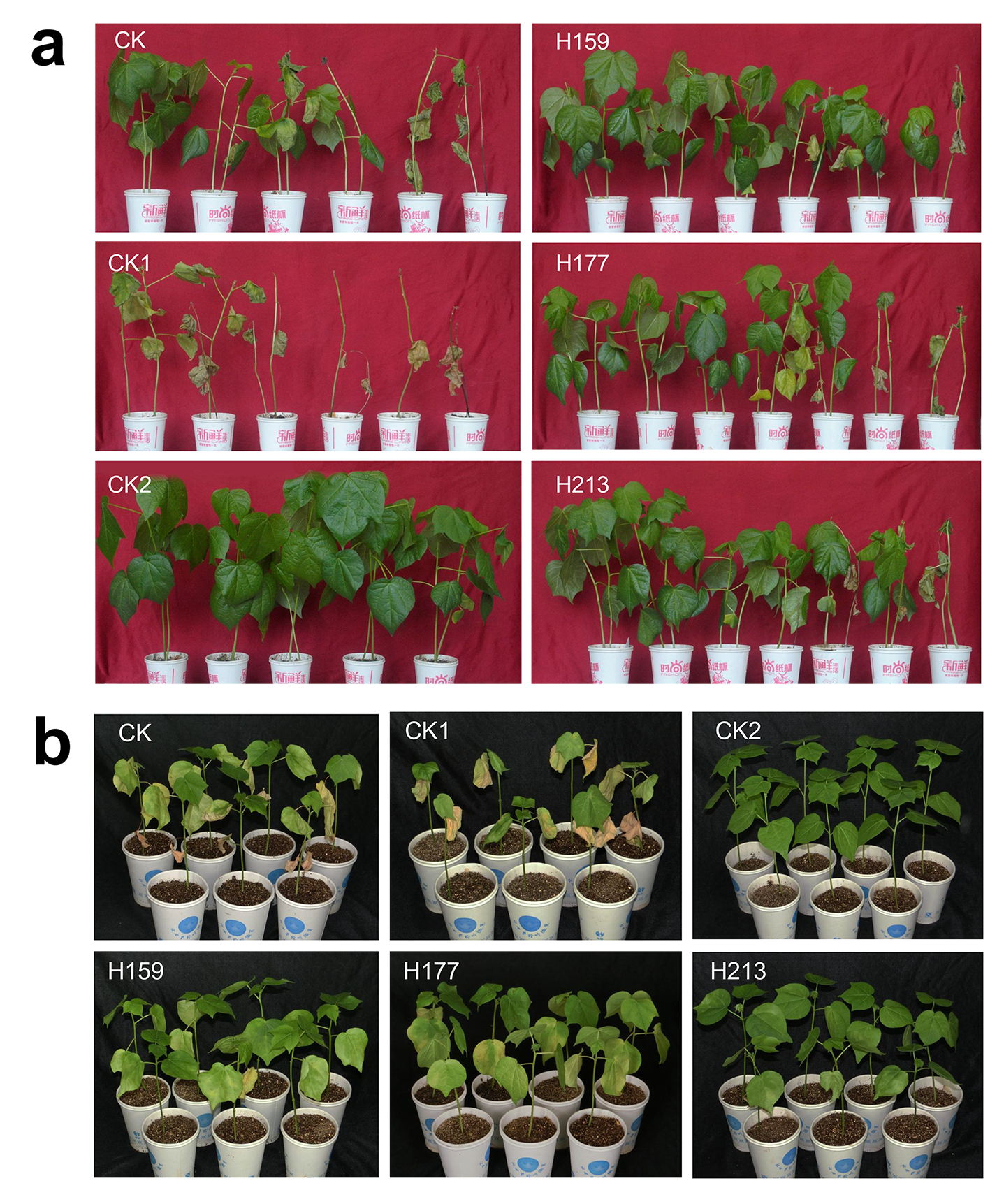
**

**Figure S1.** Phenotype of *Hcm1*-tansformed plants and parent W0 plants at 2 weeks after inoculation with *V. dahliae* isolate V991 (a) and 3 weeks after inoculation with *V. dahliae* isolate BP2 (b). CK: the parent W0 plant inoculated with *V. dahliae*. CK1: the susceptible control Junmian1 inoculated with VD; CK2: the parent W0 plant inoculated with water. Similar results were obtained from other three independent experiments.

**
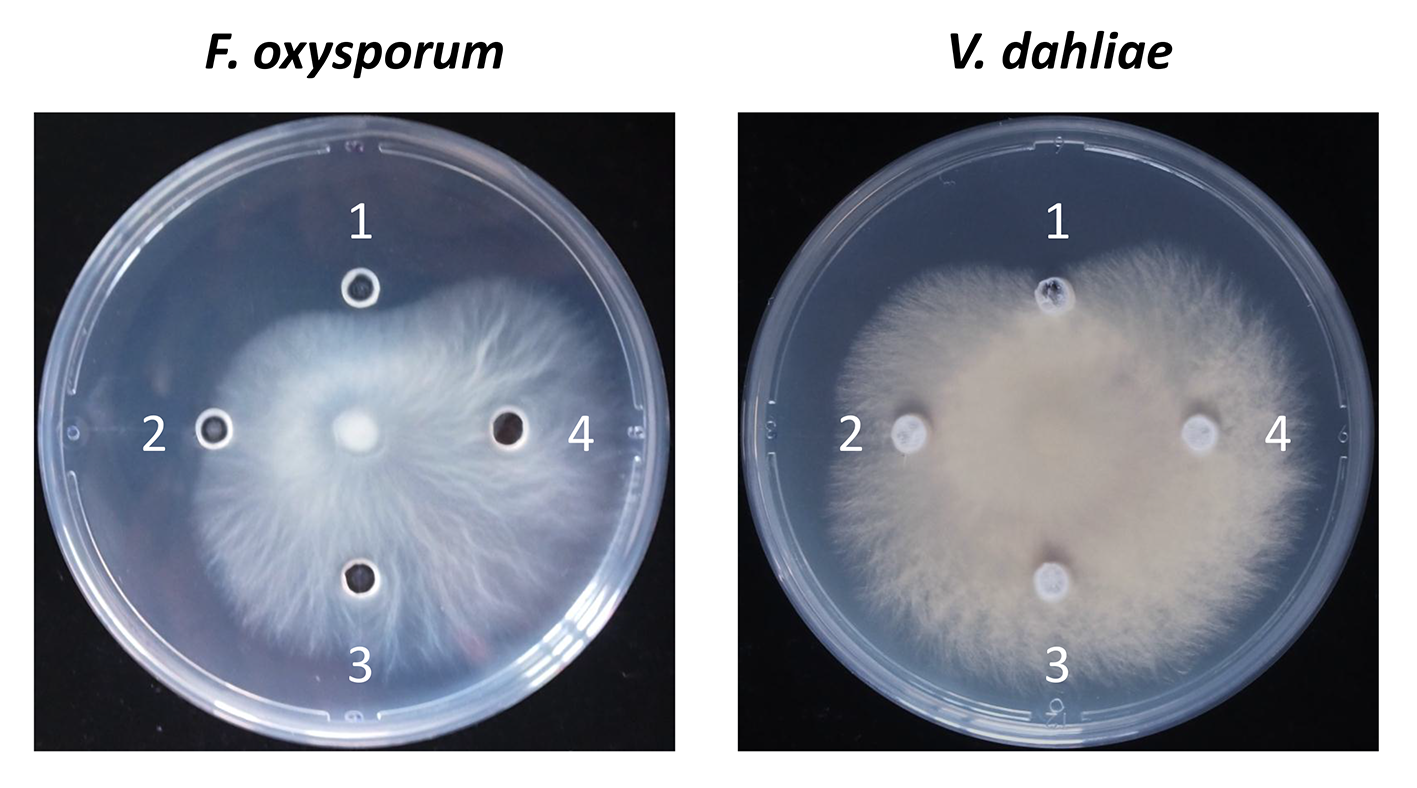
**

**Figure S2.** Antimicrobial activities of CFEPs and transgenic protein of *Hcm1* tests against *F. oxysporum* Fnj1 and *V. dahliae* V991 on CM plates. 1-4: 1mg/ml *Hcm1* CFEPs; 1mg/ml *Hcm1*-transformed protein; 200µg/ml *Hcm1*-transformed protein; and 1mg/ml parent W0 protein.


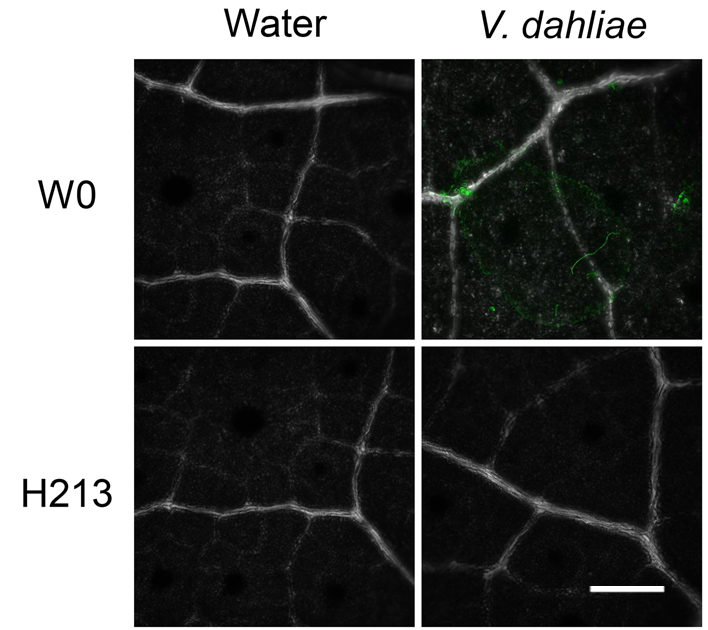


**Figure S3.** The distribution of the green fluorescent signal in first true leaves of transgenic line H213 and parent W0 plants 15 days after inoculation with *V. dahliae* harboring the GFP gene. At least 15 leaves of each line were checked in this experiment. The fluorescent signal was visualized by laser scanning confocal microscopy. Scale bar = 200µm.
